# The impact of social capital and social environmental factors on mental health and flourishing: the experiences of asylum-seekers in France

**DOI:** 10.1186/s13031-023-00517-w

**Published:** 2023-04-07

**Authors:** Maria De Jesus, Bronwyn Warnock, Zoubida Moumni, Zara Hassan Sougui, Lionel Pourtau

**Affiliations:** 1 Collegium de Lyon, Université de Lyon, Lyon, France; 2 School of International Service, American University, 4400 Massachusetts Ave, NW, Washington, DC 20016 USA; 3 Center on Health, Risk, and Society, American University, Washington, DC USA; 4 Psychologie de la Santé, Université Lumière Lyon 2, 69365 Lyon, France; 5 Santé Publique, Université Claude Bernard Lyon 1, 69100 Villeurbanne, France; 6 Pôle Recherche et Innovation, Habitat et Humanisme, 69300 Caluire et Cuire, France; 7 LEIRIS, Université Paul Valéry Montpellier 3, 34090 Montpellier, France

**Keywords:** Social capital, Social support, Social networks, Social cohesion, Asylum-seekers, France, Social environment, Mental health, Well-being, Flourishing

## Abstract

**Background:**

There is growing interest on how social capital and related social environmental factors impact overall population health and well-being. The nature of asylum-seekers’ social environment alters once they migrate to a new context and these changes influence their mental health and well-being. However, there is limited scholarship on how these social environmental factors impact the mental health, well-being, and capacity to flourish of asylum-seekers.

**Methods:**

The aim of the study, therefore, was to examine how specific social environmental factors—social networks, social support, and social cohesion at various levels (micro, meso, and macro)—influence the mental health, well-being, and capacity to flourish of asylum-seekers in France. In collaboration with a community-based organization, we used a qualitative research design to conduct 120 semi-structured interviews with asylum-seekers in France.

**Results:**

The emerging salient themes depicted how the asylum-seekers’ usual informal social networks comprised of family and friends had been disrupted since they migrated to France, which impacted their mental health and well-being. Conversely, staying connected with their informal transnational social networks via social media and developing ties with new local informal and formal social networks allowed them to receive different forms of social support, and buffered some of the negative mental health consequences. However, the lack of social cohesion due to a lack of belonging, marginalization, and current harmful migration-related policies impeded asylum-seekers’ capacity to flourish.

**Conclusion:**

While social support derived from social networks buffered some negative impacts on mental health and well-being, the overall lack of social cohesion ultimately impeded asylum-seekers’ capacity to flourish within their host communities, which was further exacerbated by harmful migration policies of exclusion within France. Introducing more inclusive policies related to the governance of migration and an intersectoral approach that views health in all policies is key to promoting social cohesion and flourishing among asylum-seekers in France.

## Introduction

There has been increasing recognition of the influence of the social environment on physical and mental health and well-being [11, 22, 24, 25, 29, 40, 41, 50, 63, 70]. The nature of asylum-seekers’ social environments change once they migrate to a new context and these changes affect their mental health and well-being [26, 33, 52]. An asylum seeker is defined as, “A person who seeks safety from persecution or serious harm in a country other than his or her own and awaits a decision on the application for refugee status under relevant international and national instruments” [55].

The concept of social capital is commonly used in social science literature to examine how the social environment affects individual and community health and well-being, as well as quality of life [1, 8, 11, 26, 35, 39, 46, 57]. Social capital is a complex concept and scholars have developed various conceptual frameworks and measures of this concept [2, 7, 18, 19, 37, 54]. An early sociological conceptualization by Bourdieu and Wacquant [7] refers to social capital as “the sum of the resources, actual or virtual, that accrue to an individual or a group by virtue of possessing a durable network of more or less institutionalized relationships of mutual acquaintance and recognition” [7]. Later, theorists Coleman [18, 19] and Putnam [54] defined social capital as the components of social organization such as networks, norms, and social trust that help in coordinating mutual benefit [18, 19, 54]. Lin’s [37] definition, on the other hand, focused on the resources embedded in social relations and social networks [37].

Asylum-seekers ability to develop new social networks, access resources, and establish a new life in a new country depends greatly on micro-level (i.e., an asylum-seekers’ formal and informal social ties), meso-level (i.e., local resources and services that facilitate asylum-seekers’ access), and macro-level influences (i.e., current migration policies) to develop their social capital [33, 48, 52]. In this study, we conceptualize asylum-seekers’ social capital as an umbrella term, encompassing three specific social environmental factors—social networks, social support, and social cohesion at various levels (micro, meso, and macro).

From a social determinants of health perspective [64, 75], there is growing interest on how these social environmental factors impact overall population health and quality of life [1, 6, 8, 22, 26, 40, 63]. However, there is limited scholarship on how these social environmental factors impact the mental health and well-being of asylum-seekers [15, 21, 25, 26, 29, 39, 41, 43, 50].

### The concept of flourishing

Expanding on the notion of well-being, Willen et al. [73] further explore the relationship between flourishing and health among asylum-seekers. For asylum-seekers, systemic inequalities and structural forms of marginalization and exclusion create health risks, impede access to needed care, and interfere with their ability to flourish. Flourishing is not just a psychological state of ‘optimal mental health,’ but an active pursuit informed by cultural expectations and social relationships, influenced by the social, political, and economic macro-level structures that shape people’s lives [73]. Extensive social capital in individuals’ lives—operationalized as strong social networks, social support, and social cohesion—positively influences their ability to flourish. Flourishing is a broad term that considers a person’s overall well-being, including life satisfaction and a sense of purpose. Flourishing, therefore, involves deeper, longer term existential goals that can intersect with and extend far beyond what transpires in clinical encounters [73].

**Fig. 1:**
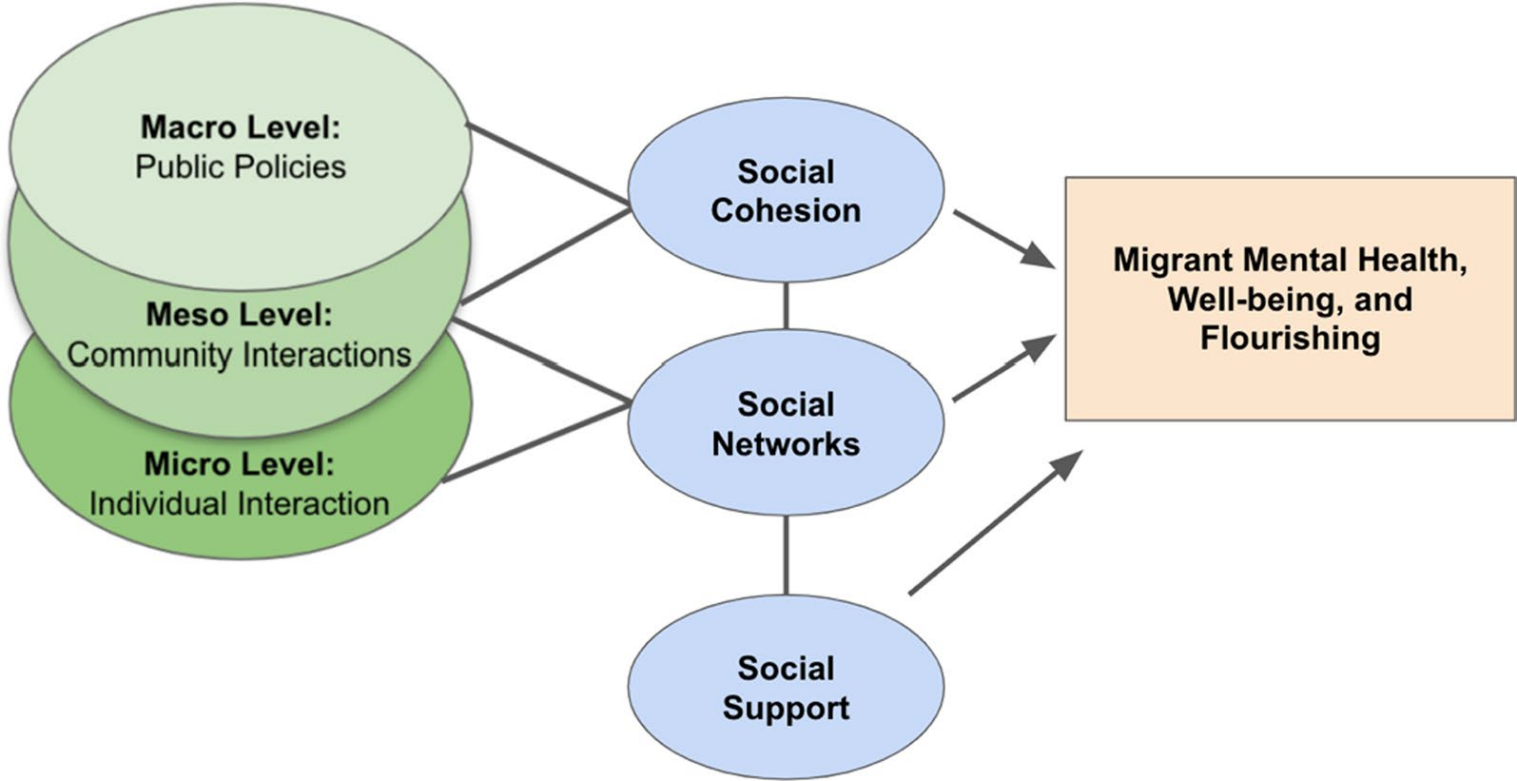
Conceptual framework: relationships among social environmental factors and asylum-seekers’ mental health, well-being, and flourishing

In the same vein, Castañeda et al. [10] calls for more advanced research initiatives which adopt “a structural approach [that] requires acknowledgment of the host of social factors and forces that affect health and operate to either include or exclude individuals and communities from adequate health care as well as from resources and experiences that foster health” [10]. There is a need for further research on how the social environment at various levels (i.e., micro-, meso-, and macro-level) impact asylum-seekers’ mental health, well-being, and flourishing. This study, therefore, fills a gap in the literature by examining how specific social environmental factors—operationalized as social networks, social support, and social cohesion at various levels (micro, meso, and macro)—influence the mental health, well-being, and flourishing of asylum-seekers in France (Fig. 1).

## Social environmental factors

Asylum-seekers undergo intricate adjustments in the host country, which stem beyond individual-level changes and involve a “complex and often protracted process of negotiation with social structural, political, and economic forces” [10]. For the purposes of this paper, we will focus on the impact of social networks, social support, and social cohesion on asylum-seekers’ health, well-being, and flourishing. Social networks are defined as ties between two or more individuals based on a “common thread of interest” or “relations of kinship, friendship… [or] shared community origin,” as well as with external contacts that do not identify as asylum-seekers themselves [51]. Social networks can manifest in different forms due to different incentives in forming networks [23]. Therefore, in extant literature, asylum-seeker social networks have been described according to a combination of different descriptive characteristics: formal/informal, vertical/horizontal, heterogenous/homogenous, weak/strong as well as transnational/local network ties. Table 1 outlines a typology of social networks and their respective definitions.

**Table 1:** Typology of social networks among asylum-seekers

Formal versus informal networks [28, 35, 72]	Formal: ties established within the workplace or within institutions (e.g., medical providers, social workers, etc.)	Informal: ties formed among family, personal friends, and acquaintances
Vertical versus horizontal networks [56, 58]	Vertical: bonds formed with members of different socio-economic statuses, interests, backgrounds or other categories	Horizontal: bonds formed with members of similar socio-economic statuses, interests, backgrounds or other categories
Heterogenous versus homogeneous networks [11]	Heterogeneous: ties composed of people from different cultural backgrounds and/or asylum-seeker status	Homogeneous: ties between individuals of similar cultural backgrounds and/or asylum-seeker status
Weak vs. strong networks [11, 58, 72]	Weak: ties that do not stem into further, deeper relationships	Strong: composed of individuals with strong and everlasting relationships
Transnational networks versus local networks [23, 57]	Transnational: relationships between members across country borders, which are frequently between asylum-seekers and relatives/kinships from their home country	Local: relationships between members within the same country/community, can be relationships among asylum-seekers and relationships between asylum-seekers and other members of the community
Dense versus loose networks [4, 67]	Dense: Networks with a large quantity of social ties, which tend to be small and stable communities with few external contacts and a high degree of social cohesion	Loose: Networks with a small quantity of social ties, which tend to be large and unstable communities that have many external contacts and exhibit a relative lack of social cohesion

Social networks at a micro-level refer to the effects of individual relationships or interactions on health and well-being [51]. At the meso-level, social networks can be assessed by acknowledging the interconnectedness of health among socially tied individuals at the community level [63]. Meso-level social networks are important to asylum-seekers in terms of promoting social cohesion and informing asylum-seekers of health information resources, which impact asylum-seeker health [23, 50]. A study among sub-Saharan African asylum-seekers in France demonstrated that these meso-level social networks and a community-based outreach approach impacted the engagement of the asylum-seekers in health prevention programs [29].

Social support is derived and fostered from social networks, which impacts health, well-being, and quality of life [5, 23, 57]. Asylum-seekers provide and obtain different types of social support such as emotional support, financial support, and instrumental support from their social networks [17, 50, 57, 63]. On a meso-level, social support achieved through social networks within a tight-knit community can have a positive impact on mental well-being among asylum-seekers despite the challenges of migrating [3, 25, 28].

Social cohesion is a measure of “positive social relationships” [41] and the extent of connectedness among groups, which influences health through its role in promoting health-related behaviors and increasing access to services and resources [6]. The concept of social cohesion at the meso-level has two main dimensions: the sense of belonging to a particular group and the relationships among members within the group itself [42]. Alternatively, social cohesion at the macro-level is understood as a process “by which the whole society, and individuals within, are bound together through the action of specific attitudes, behaviors, rules, and institutions, which rely on consensus rather than pure coercion” [31]. One approach to measuring social cohesion is by people’s perceptions of how social groups get along with each other in their local area and by levels of trust in local institutions (such as the police) (e.g., [59]. A lack of social cohesion such as perceived discrimination can have a negative effect on health and well-being including negative mental health effects (i.e., anxiety and depressive symptoms) [14, 36, 49, 61, 62, 74]. 

Conversely, a macro-level approach focuses on the potential of inclusive policies in fostering social cohesion and specific actions to promote anti-discrimination and mutual understanding while countering xenophobia [34]. From this macro-level perspective, social cohesion provides a policy goal which aims to achieve positive outcomes such as social integration and equity for all members of a society [65]. For example, the Organisation for Economic Co-operation and Development (OECD) report (2011) characterizes social cohesion as, “a cohesive society [that] works towards the well-being of all its members, fights exclusion and marginalization, creates a sense of belonging, promotes trust, and offers its members the opportunity of upward mobility” [45]. From this perspective, social cohesion can be used as the basis measurement of various social outcomes to inform and check policy intentions [65]. This consists of a multi-dimensional approach that assesses how policies across key sectors, such as economics, labor, education, environment, civic participation, and social protection, can enhance social cohesion. The objective is to help countries improve their economic and social policies in a way that fosters social inclusion, social capital, and social mobility [43, 45, 47, 50, 70].

### Context of asylum-seekers

Asylum-seekers face many challenges, including a lack of economic and democratic participation, as well as employment restrictions, which create a reliance on non-state and state actors for basic needs (e.g., allowances, food, housing). In addition, stricter healthcare policies within France in recent years have created more barriers for asylum-seekers in need of health services, including mental health services. The 2019 healthcare reform policy known as ‘*L’aide médicale de l’État* (AME),’ or the Federal Medical Assistance, now requires asylum-seekers to complete a 3-month “délai de carence” (waiting period) in France prior to accessing basic healthcare coverage [66]. New restrictions on the type of care available were also introduced, for example, excluding psychiatric support. Perreira and Pedroza [50] refer to these migration-related legal structures that limit access to employment and healthcare as “policies of exclusion,” which include an array of federal, state, and municipal laws and administrative practices that hinder asylum-seeker integration [50].

## Methods

### Study design and participant recruitment

This study was part of a larger, 2-year mixed methods research project in collaboration with a community partner, *Habitat et Humanisme* in France. This organization provides asylum-seekers with access to accommodation centers where they can obtain social, administrative, legal, and integration support. Approval for this study was obtained from the Institutional Review Board of the Université de Lyon. Between September 2019 and August 2021, we began our field research with asylum-seekers. Our field data collection was temporarily halted between March and May 2020 due to the COVID-19 pandemic and resumed in June 2020 following the lockdown in France.

We contacted 16 accommodation centers that serve asylum-seekers in the southeast-central region of Auvergne-Rhône-Alpes and in the north-central region of Île-de-France. Twelve of the centers were affiliated with *Habitat et Humanisme*. Our partnership with *Habitat et Humanisme* facilitated our entry into the centers as the community partner had already established a strong level of trust with the staff and the participants. We went to each of the centers and held study information sessions to describe the study and the eligibility criteria (i.e., at least 18 years of age; asylum-seeker status; and currently residing in France) and answer any questions from potential participants. We also emphasized that participation was voluntary. Following the information session, interested participants enrolled in the study.

All the participants resided in the accommodation centers with 3 or 4 asylum-seekers per shared room. The centers varied in terms of size, accommodating between 75 to 450 asylum-seekers per center. The centers that were in urban settings typically provided a shared kitchen and bathroom for the groups of asylum-seekers, while the more remote centers offered a collective kitchen and communal sanitary facilities for all the residents. The urban centers provided the asylum-seekers with easier access to public transportation, services, and amenities, while the asylum-seekers in more remote areas were less mobile and more isolated from services and amenities.

The authors first employed a qualitative, inductive research design, which is concerned with an emic, idiographic approach to research, and subsequently used a deductive design [71], organizing the salient themes into the micro-, meso-, and macro-level framework of each of the social environment factors. A semi-structured interview guide was developed to ensure consistency across interviews. This article presents data from the interviews that we conducted with a total of 120 asylum-seekers. Given that initial trust had been established with the community partner, there was a strong level of participation across the centers.

Participants were first asked open-ended questions related to their experiences with the asylum-seeking process and their daily lives as they waited for an outcome. For example, what has the asylum process been like for you thus far? What is your day-to-day experience where you live and with those around you? Subsequent interviews were conducted with more asylum-seekers to better understand the emergent issues. New questions were therefore added to the guide to probe into the initial themes such as, How do your relationships with specific people impact your mental health and well-being? Tell me a story about an important relationship for you to help me better understand how this relationship affects your well-being. How would you describe your current social relationships? Have they changed since you arrived in France, and if so, how have they changed? How do your relationships with others impact your mental health and well-being? What are some of the mental health impacts you have experienced? To what extent, if any, do specific policies affect your social relationships? Do these policies harm or facilitate your social relationships? How so?

Participants were interviewed in a private office at each of the centers at a day and time of their convenience. Each interview lasted about 90 min. The first, third, and fourth authors conducted all the interviews. These authors have a background in public health and psychology and had extensive training and experience with the data collection and analysis techniques used. We obtained informed oral consent from each participant prior to the interview rather than written consent due to the sensitivity in obtaining signatures from an asylum-seeker population. The interviews were conducted in English or French, and an interpreter was used for the participants who stated that they preferred to do the interview in their native language. Each interview was recorded and transcribed into English. Pseudonyms were used to protect the participants’ confidentiality. The researchers conducted an iterative process of data collection and analysis and held weekly debrief meetings to reflect on the data, what they were learning, and their experiences with the interviews.

### Data analysis

We began with an inductive analytic process whereby we generated codes based on the data themselves, and subsequently, progressed to a deductive process where we applied a set of codes based on the micro-, meso-, and macro-level framework of each of the social environment factors (social networks, social support, and social cohesion) to the data [69]. Qualitative thematic analysis was used to analyze the interview data and we adapted the steps as outlined by Luborsky [38]:

1. *Selecting the unit of analysis:* we chose the phrase as a separate entity of meaning.
2. *Creating and defining the codes:* The coding began as an iterative, data-driven, and inductive process. We followed an iterative process of independently reading the interview transcripts and using a line-by-line coding method, we each added emergent codes from the data to develop a codebook (ATLAS.ti version 8.4). We modified the codebook as needed during the analytic process. Subsequently, we used a deductive process given that the emergent data revealed the appropriateness of applying a micro-, meso-, and macro-level framework of each of the social environment factors to the data. During weekly online debrief meetings, we discussed points of disagreement and modified the codes as needed.
3. *Identifying themes*: We located patterns in the codes and identified themes under specific codes. Themes were derived directly from the participants’ words. This was an interpretive process where we searched for meanings at the level of coding as well as how factors at different levels influenced each other.
4. *Assessing validity*: To ensure interpretive validity, the lead researcher took the research findings back to the participants. This involved a process of member-checking, whereby we verified the data and the interpretations with the participants. Some of the participants were no longer present at the center. Thus, member-checks were a mix of original and new participants.

## Results

Table 2 summarizes the sociodemographic characteristics of the study sample. Most participants were male (78%) and the median age for the sample was 26 years old, which represents the asylum-seeker population in France. Approximately 83% of the sample had a primary or secondary level education. While 64% of participants had an intermediate level of French language comprehension, the same percentage of participants (64%) had only a beginner level of oral expression in French. Approximately 72% of the sample was from an African country, while one quarter (28%) was from Afghanistan. The sociodemographic data for this study is comparable to similar studies with asylum-seekers in France [9, 20]. The findings depict three domains—social networks, social support, and social cohesion—and the salient themes corresponding to each domain at the micro-, meso-, and macro-levels in addition to their impact on the asylum-seekers’ health, well-being, and ability to flourish from the perspectives of the asylum-seekers themselves (Table 3). Pseudonyms are used for all the participants.

**Table 2:** Sociodemographic characteristics of study sample (*N* = 120)

Characteristic	Number of participants (%)
Gender, n (%)	
Male	94 (78.33)
Female	26 (21.67)
Age (years), median	26
Education, n (%)	
Primary	45 (37.50)
Secondary	54 (45.00)
Post-secondary	13 (10.83)
Little or no schooling	8 (6.67)
Level of comprehension in French, n (%)	
Beginner	38 (31.66)
Intermediate	77 (64.17)
Advanced	5 (4.17)
Level of oral expression in French, n (%)	
Beginner	77 (64.16)
Intermediate	38 (31.67)
Advanced	5 (4.17)
Country of origin, n (%)	
Afghanistan and Pakistan	36 (29.99)
Western African countries	57 (47.51)
Eastern African countries	13 (10.84)
Central African countries	8 (6.66)
Northern African countries	6 (5.00)

**Table 3:** An overview of the salient themes by each social environmental factor at the micro-, meso-, and macro-levels and impact on mental health, well-being, and ability to flourish

	Social networks	Social support	Social cohesion
Micro-level	Theme 1a. I miss my wife and kids. I am worried for their well-being. I feel depressed and cannot sleep well Theme 1b. I try to talk to my mom every week on WhatsApp. This helps me feel less depressed	Theme 4. I can count on my friend here at the center. I am depressed. It is hard to wait and not know what will happen to me in my future. But I look to him so I can keep going each day. He helps me feel less depressed	Theme 7. I do not feel comfortable leaving the center. I am invisible here in France and do not feel like I belong. I feel alone and depressed
Meso-level	Theme 2. I know I can count on the people who work here. I am well accompanied for the administrative procedures. These people help me deal with my anxiety	Theme 5a. I like when volunteers from the area and the university come to spend time with us. I feel heard. It also helps me deal with my stress of being in my situation. I can talk about other things with them Theme 5b. No one is available to help me. I go to the appointment with the staff member, I explain my concerns, but there is very little help they offer me. It makes me feel more anxious	Theme 8. No one approaches me here in France. They do not see me. They do not know me. I feel lonely and depressed at times. The French people mobilized and were very vocal about wanting us out of here. Some extremists even posted a sign outside where we lived demanding that we leave
Macro-level	Theme 3a. I am completely apart from the French people. I am marginalized. I need the vital things: to go to work. I do not want the government to give me money. I want to go to work. I feel I spend a lot of time doing nothing. Staying all day and doing nothing, plays with your mind. You get depressed or angry. There are days I am feeling depressed and then I am mad. This is not good for my health. I want to do more with my life and be healthy Theme 3b. There are older people and younger people who are here to help us. The instructors and volunteers help us learn French. They are our connection to learning more about French language and culture and to feeling less lonely	Theme 6. Going through the asylum-seeking procedures […] and policies are not there to help me and not getting the government supports has been a really hard struggle. I am completely overwhelmed and depressed. I do not see the end nor a way to move forward with my future	Theme 9. I have no certainty about my future in France right now because I lack papers here. I can be deported. I cannot work without papers. I have little security. With papers, I will have more security. I can work and then find a path to bring my 9-year-old daughter here from Mali. We can be together as a family again

### Social networks

#### **Theme 1a**

I miss my wife and kids. I am worried for their well-being. I feel depressed and cannot sleep well.

Informal social networks at the micro-level are comprised of relationships with family members and friends. These social networks include transnational relationships across country borders, often between asylum-seekers and relatives “back home” or in transit countries. All the participants, like Abdul-Azim, a 28-year-old male asylum-seeker from Afghanistan who reported theme 1a, stated how their relationships with important individuals in their social networks had been disrupted since they migrated to another country and how these disruptions contributed to depression, social isolation, and insomnia. Similarly, a 25-year-old female asylum-seeker from Nigeria, Ginika, shared how her main source of support was gone and how she was still grieving in the post-migration context, “I lost my husband. I am still grieving here. I am very depressed.” Another asylum-seeker from Guinea, 23 years of age, spoke about how he was denied asylum in Austria and, as a result, experienced a disruption of his friend network when he fled to France, which led to depressive symptoms and social isolation, “I was in Austria for 4 years and had my life there. I miss my friends there, more than my own family. They were my family. There are days I am so depressed; I just stay in my room all day.”

#### **Theme 1b**

I try to talk to my mom every week on WhatsApp. This helps me feel less depressed.

Although all of the asylum-seekers’ social networks had been disrupted in the post-migration context, approximately half of the participants were able to stay connected to their informal and transnational social networks using technology, like Abdul, a 23-year-old male from Afghanistan expressed in theme 1b above, which helped them cope with their depressive symptoms and social isolation. Similarly, Seydou, a Malian 26-year-old male asylum-seeker, expressed, “*I call my wife to know how she and our kids are doing. This helps me feel less depressed and isolated*.”

#### **Theme 2**

I know I can count on the people who work here. I am well accompanied for the administrative procedures. These people help me deal with my anxiety.

At the meso-level, many of the participants spoke about how formal social networks in France with which they interacted helped them to alleviate some of their anxiety and stress. These networks are comprised of individuals outside the asylum-seeker circle and are vertical and heterogeneous in nature (refer to social network typology in Table 1). Most of the participants, like Abdirahim, a Somalian 20-year-old male described in theme 2 above, stated how they counted on these formal networks comprised of social service workers, medical providers, and volunteers to assist them with their different needs (e.g., filing paperwork, learning French, making medical appointments), thereby helping them cope with their anxiety and stress. In the same vein, Abdul-Ali, a 26-year-old male Afghan asylum-seeker expressed, *“If I want to go to the doctor, the social workers can help me get an appointment. I feel less stressed when the social workers and doctor help me.”* The asylum-seekers were able to obtain social support as well as important resources and information through these networks. In addition, these formal networks promoted a sense of social cohesion for asylum-seekers, which also improved their ability to cope with symptoms of anxiety and stress. Although many participants felt that they could rely on staff from the center and other formal social ties, this perceived social support was not generalized to the wider French society due, in part, to a lack of health-promoting policies.

#### **Theme 3a**

I am completely apart from the French people. I am marginalized. I need the vital things: to go to work. I do not want the government to give me money. I want to go to work. I feel I spend a lot of time doing nothing. Staying all day and doing nothing, plays with your mind. You get depressed or angry. There are days I am feeling depressed and then I am mad. This is not good for my health. I want to do more with my life and be healthy.

At the macro-level, most of the participants, like Oumou, a 22-year-old male asylum-seeker from Mali described in the third theme, stated how a lack of permission to work policy for asylum-seekers contributed to feelings of marginalization, depression, anger, and an inability to flourish. Asylum-seekers’ inability to work also promoted misperceptions of asylum-seekers by the French public. Amin, a 24-year-old male asylum-seeker from Sudan, described how the French did not understand why asylum-seekers were unable to work, stating, “French people ask me, ‘Why don’t you work and do something with your time?’ They think asylum-seekers are lazy! They see us here [asylum-seeker center] or wandering through the streets. They have no idea that we are not allowed to work! It is not a voluntary choice. I wish I could work. I cannot work because I have no papers. We are all here waiting and wasting time away every day. I cannot get to my goals.”

Another 24-year-old asylum-seeker from Afghanistan spoke about how the inability to work blocks his ability to flourish and contributes to his depression, “I had plans to work, rebuild a life for myself and eventually, my family […] I left the violence and problems in my country and now I have a new set of problems here. I lost my sense of purpose. I can’t work and build a future. I am depressed.” An asylum-seeker, a 20-year-old male from the Ivory Coast, Kouakou, also explained how these restrictive laws harmed asylum-seekers, “They do not permit us to work. We wait and wait, with no answers. We cannot live well without being able to work. We can’t integrate. We are separate from everyone else. Our migration status hurts us. These asylum policies here hurt us. If we worked, we could interact with more French people. We could practice French and feel more like we are part of this society.” The macro-level policies excluded asylum-seekers and contributed to their sense of marginalization, depressive symptoms, and inability to flourish. Additionally, it promoted xenophobic and discriminatory attitudes from the larger public toward asylum-seekers.

#### **Theme 3b**

There are older people and younger people who are here to help us. The instructors and volunteers help us learn French. They are our connection to learning more about French language and culture and to feeling less lonely.

Conversely, the French language policy that provides asylum-seekers access to free formal French classes on a weekly basis facilitated their access to formal French social networks, their ability to communicate in the dominant language as well as navigate French culture and social norms. As many participants recounted, including Djoëlle, a 24-year-old female asylum-seeker from the Democratic Republic of the Congo who reported theme 3b, the opportunity to take French classes allowed them to form relationships with French instructors, volunteers, and other asylum-seekers in the class, which decreased their sense of loneliness. Many of the asylum-seekers reported how their social networks facilitated access to social support, as described in theme 4.

### Social support

#### **Theme 4**

I can count on my friend here at the center. I am depressed. It is hard to wait and not know what will happen to me in my future. But I look to him so I can keep going each day. He helps me feel less depressed.

The horizontal social networks above-mentioned provide opportunities for many of the asylum-seekers to receive various types of social support (i.e., emotional support, etc.) at the micro-level from their informal relationships with other asylum-seekers, thereby reducing their stress and depressive symptoms, as described in theme 4 by Moussa, a 23-year-old male asylum-seeker from Guinea. Several asylum-seekers spoke about receiving financial support from fellow asylum-seekers when they were in dire need of support. As Abdirahim, a Somalian 20-year-old male stated, “Last month, it got hard to buy things I need. It was very stressful. The money is so little. I got help from my friend here […] praise ne to Allah, he helped me!” Mariame, a 28-year-old female from Eritrea, also recounted how she relied on her African asylum-seeker friends who she called her “sisters” for informational support, which reduced her stress, “I talk to my African sisters every week. She shares important information with me like where to go for food, which doctor to go see, how to send money home, things that are helpful for me […] Now I am less stressed.” The asylum-seekers described how receiving upport from fellow asylum-seekers diminished their levels of stress and depression.social s

#### **Theme 5a**

I like when volunteers from the area and the university come to spend time with us. I feel heard. It also helps me deal with my stress of being in my situation. I can talk about other things with them.

At the meso-level, asylum-seekers, like Abdul-Ali, a 26-year-old male Afghan asylum-seeker who stated in theme 5a, shared how receiving social support from local food banks, clinical encounters, volunteers in the community who visited the center, and some of the staff members at the center, reduced their anxiety and stress levels. Similarly, Djoëlle, a 24-year-old female asylum-seeker from the Democratic Republic of the Congo, reported, “I know I can go to the place down the block, and they will give me some food donations. I can get out of the center and feel less anxious.”

#### **Theme 5b**

No one is available to help me. I go to the appointment with the staff member, I explain my concerns, but there is very little help they offer me. It makes me feel more anxious.

Conversely, about a third of the participants spoke about how they received very little social support from formal social networks, which contributed to their anxiety. As reported in Theme 5b, Tawab, a male asylum-seeker from Afghanistan, recounted receiving little support from center staff. Fatouma, a 28-year-old female asylum-seeker from Mali, expressed a similar experience, connecting it to a lack of supportive policies, “We cannot really count on the staff. They are here to do a job but there are few resources for them to offer us. Their hands are tied because the State does not permit more resources or services. The government policies related to asylum-seekers are not helping us. They make it harder for us. I am so worried.” Other asylum-seekers also understood how a lack of social support was influenced by adverse government policies, as described in theme 6.

#### **Theme 6**

 Going through the asylum-seeking procedures […] and policies are not there to help me and not getting the government supports has been a really hard struggle. I am completely overwhelmed and depressed. I do not see the end nor a way to move forward with my future.

At the macro-level, many asylum-seekers described how a lack of formal government social support through beneficial policies for asylum seekers contributes to a cycle of anxiety and/or depression as well as a sense of uncertainty about the future, all of which hinders their ability to flourish, as described by Abdul-Azim, an Afghan asylum-seeker in theme 6. An example of a policy that has thwarted their ability to develop long-lasting social networks with other asylum-seekers as well as French people from whom they have received social support is a national dispersion policy that often assigns asylum-seekers to more remote areas. Many of the participants described how being uprooted from where they are and shuffled to other parts of France or elsewhere contributes to their depressive symptoms and ability to flourish. As Fikru, a 23-year-old male asylum-seeker from Eritrea explained,

As soon as you are feeling a little connected to the people that live in the same place and the French volunteers who come from the outside, it is time to go to a different place. A few of us will be lucky and get refugee status, others will end up in another center far away, others will be deported, and others will go onto the streets with no stable place. We end up losing touch. It is hard to form long-standing connections with some of my friends here. They already told us that this center is shutting down in about a month. I am depressed and feel blocked from constructing a good future for myself.

Another policy which asylum-seekers also described as having similar detrimental mental health impacts, in terms of depressive symptoms and anxiety as well as their ability to flourish, is the Dublin regulation, which refers to the rules about which country should assess an asylum-seeker’s application for international protection. At least half of the asylum-seekers in the study had been in another European country prior to arriving in France and had been uprooted from that country. Many were now under the “Dublin procedure” in France and it was still unclear which country within the European Union (EU) was responsible for examining their application for asylum. Until confirmation of which EU Member State was responsible for their application, the French authorities did not consider the details of their asylum application, which delayed the whole asylum process even more. As a result, these asylum-seekers were in limbo, not knowing where their future lay. As Bahiri, a 24-year-old male Afghan asylum-seeker who had been in Germany prior to coming to France and was denied asylum in Germany described:

I felt I wasted 5 years of his life. When I was in Germany, I played soccer every day. I went to school. I learned the language. I had friends. I had a life. I am in France now and don’t feel I can start all over. What if the same thing happens again and then I cannot stay here? Then what? It is harder to work towards any future goals. I am just waiting and wasting time. I cannot work here. I cannot go to university. I am not well. I am worried and anxious all the time.

In addition to a lack of macro-level social support, which contributed to anxiety among asylum-seekers, the participants also spoke about how their perceived lack of social cohesion within their new communities in France impacted their sense of belonging and mental health.

### Social cohesion

#### **Theme 7**

I do not feel comfortable leaving the center. I am invisible here in France and do not feel like I belong. I feel alone and depressed.

Although the majority of asylum-seekers felt safe within the confines of the asylum-seeker center or their personal living space, this was not the case outside of the center or their living space. At the micro-level, most of the participants expressed a lack of social cohesion with approximately two-thirds of the asylum-seekers, like Amin, a 24-year-old male Sudanese asylum-seeker described in theme 7, reporting that they did not feel like they belonged to French society. Many asylum-seekers attributed their lack of belonging to the fact that they could not work, go to school, or contribute meaningfully to French society as they awaited their asylum cases to be adjudicated. Nawaskhan, a 25-year-old male asylum-seeker from Afghanistan stated, “I am sad when I remind myself that I am in an unknown land where I do not speak the language. I lost a lot: my husband, my land, my familiarity…I am alone here. No one here really knows me or cares about me. If I get sick, who will I turn to? Where will I go? I feel depressed.”

#### **Theme 8**

**Fig. 2:**
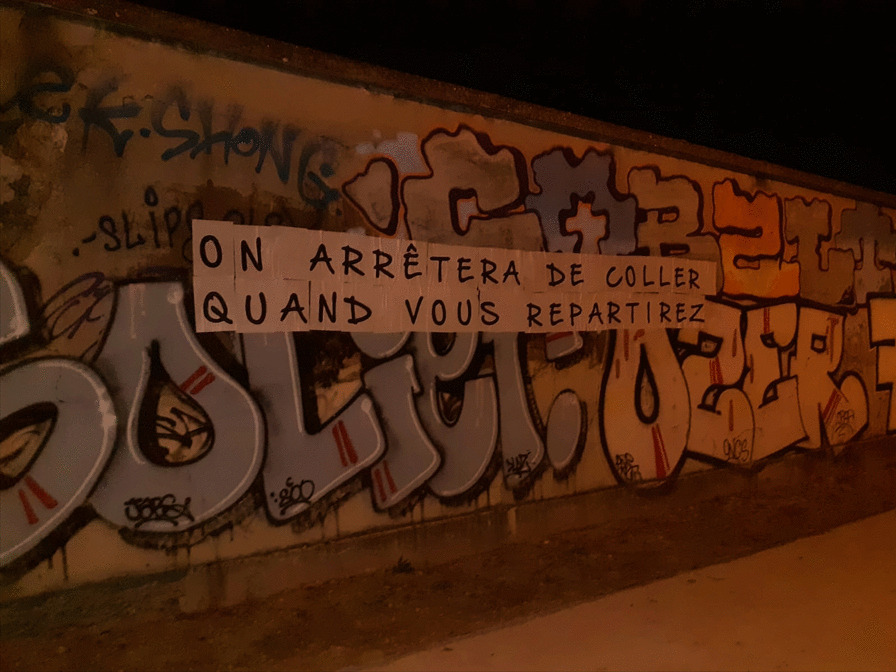
Sign stating “We'll stop sticking [these signs] when you leave” outside asylum-seeker center in Lyon, France. (copyright: Author 1)

No one approaches me here in France. They do not see me. They do not know me. I feel lonely and depressed at times. The French people mobilized and were very vocal about wanting us out of here. Some extremists even posted a sign outside where we lived demanding that we leave [Refer to Fig. 2].

At the meso-level, along with the experience of having been uprooted, many asylum-seekers experienced a lack of social cohesion in the host community, which contributed to their loneliness and depression, as expressed by Abdirahim, a 20-year-old Somalian asylum-seeker in theme 8. Similarly, Fikru, a 23-year-old male asylum-seeker from Eritrea, also reported feeling like an “outsider” stating, “I do not feel included. I have few connections with any French people. They do not see me. I have no native French friends. I feel lonely.”

All the asylum-seekers also expressed how they felt misunderstood or negatively perceived by French citizens, which gave rise to feelings of anger and sadness. Mohamed, a 27-year-old male Congolese asylum-seeker expressed a lack of understanding from neighbors, “We are blamed for all the bad things that happen in the neighborhood even if it is not us. Last week the neighbors complained that the asylum-seekers in the center left dog poop on the sidewalk. How could it be the asylum-seekers? We do not even have a dog! This makes me angry!” Another asylum-seeker, 24-year-old Hasina from Madagascar, reported a similar sentiment, “We sometimes read in the paper about terrible things that some people in our country have done. I do not represent my entire country. People here judge me and other asylum-seekers for these actions. This brings sadness to me. Why do we have to pay for all the negative things people in our country do?”.

Another asylum-seeker from Guinea also expressed a lack of social cohesion within the community and a need to be known by French citizens and the French government,

What I need the most is for people to understand my situation, for the government to understand my situation. I feel a certain sadness and even anger over the fact that France does not see my country as a very dangerous country. They blame us for what is going on. There is a lack of understanding of our reality. We are not fleeing misery because we can manage misery, even in France, there is misery. But what we are fleeing is violence.

Other asylum-seekers expressed a need for French citizens to make a link between what is happening now with their country and what took place in history, in terms of the colonization by France. An asylum-seeker from Senegalese put it this way, “We need to understand the roots of causes. We need to understand that there is a history too between France and its former colonies. People do not understand this. This bothers me given the situation in Africa, and what is happening in our countries there and the role France plays there.” In addition to a lack of meso-level social cohesion, the participants spoke of the effects of a lack of macro-level social cohesion, including the inability to gain stability in their lives and to flourish.

#### **Theme 9**

I have no certainty about my future in France right now because I lack papers here. I can be deported. I cannot work without papers. I have little security. With papers, I will have more security. I can work and then find a path to bring my 9-year-old daughter here from Mali. We can be together as a family again.

At the macro-level, all the asylum-seekers spoke about the impact of migration-related policies and how a lack of refugee status affected their sense of security and stability regarding their future. Many of the asylum-seekers, like Dejeneba, a 37-year-old male asylum-seeker from Mali described in theme 9, stated that not having documents that showed refugee status, commonly referred to as ‘papers,’ decreased their sense of security and capacity to flourish in their new communities. In a similar vein, Nawaskhan, a 25-year-old male asylum-seeker from Afghanistan stated, “Without papers, I have no identity. People do not see me. Having refugee status would allow me to be safe, form a family …To somehow have a normal life.”

Apart from the effects that spur from a lack of legal documentation showing refugee status, other asylum-seekers spoke about how current migration policies created barriers for asylum-seekers, which took a toll on their mental health. As Mohamed, a 27-year-old male Congolese asylum-seeker expressed,

Even though some people have the right to protection and need protection, they are rejected. They are blocked from being able to move forward with their lives in France. It is depressing to live like this with no pathway to a future. Today’s policies have not evolved to reflect the current realities related to migration and why we are migrating to France and other European countries. Many of us are fleeing violence. There is no protection from the government in our countries. In some cases, they are the perpetrators or sponsors of the violence.

Another asylum-seeker, a 24-year-old male from Nigeria, expressed frustration and anger with the current migration policies, “I do not understand these policies! France signed onto a treaty for the protection of asylum-seekers. France is a humanitarian country. How could the policies then be so inhumane towards us asylum-seekers? We are not here to feast on French baguettes. We are fleeing violence […] We need safety and protection.” Similarly, a 24-year-old asylum-seeker from Afghanistan described the migration policies as “arbitrary” and expressed his anger, “These laws seem to contradict what is happening in reality. France and other European States say that Afghanistan is a safe country. It is not on the list of dangerous countries. We can now live peacefully in Afghanistan. There is conflict still there and it is unsafe. They accept Syrians more easily. Why not us? It is arbitrary. I am angry with this situation!” Overall, the macro-level policies negatively impacted the capacity of these asylum-seekers to flourish in their host community.

Although we unpacked the influence of each of the social environmental factors—social networks, social support, and social cohesion—on the mental health and ability to flourish of the asylum-seekers, the participants themselves understood how each of these aspects related to one another based on their lived experiences. For example, disrupted social networks and experiences of uprootedness meant a lack of social support, less certainty about the future, and less social cohesion and belonging. These experiences contributed to their depressive symptoms, anxiety, loneliness, anger, and sleep quality, as well as their overall ability to flourish in France.

## Discussion

This study unpacked the relationships between specific social environmental factors (social networks, social cohesion and social support) on asylum-seekers’ mental health and well-being as well as their capacity to flourish. We found that a disruption of social networks and a lack of social support and/or social cohesion leads to poor mental health (e.g., depressive symptoms, anxiety, loneliness) and well-being for asylum-seekers within France. These findings are in line with previous studies that demonstrated how perceived discrimination can have effects on mental health, including symptoms of anxiety, depression, and post-traumatic stress disorder [14, 36, 61, 74]. Conversely, we found that asylum-seekers that were a part of both formal and informal, local and transnational social networks were able to derive various forms of social support from these social ties which positively impacted their mental health and well-being, these findings also align with previous literature [5, 17, 23, 50, 53, 57, 63]. While some asylum-seekers rely on local and transnational social networks and these various forms of social support, which positively impact their mental health, ultimately, the lack of social cohesion as a result of macro-level policies negatively impact their ability to flourish in their host community.

Many asylum-seekers relied on social support and social networks to survive daily. The various forms of social support derived from their different social networks elevated their mental health and well-being. However, there was a lack of social cohesion needed in the long-term within their social networks and within the larger French society. Thus, it was difficult for them to flourish and experience security about their futures. Overall, many of the asylum-seekers spoke of a lack of belonging to French society, which is a key dimension of social cohesion [42, 45]. These asylum-seekers described how they had few connections with French individuals and felt misunderstood or perceived negatively by French society. These experiences can be interpreted through Holmes and Castañeda's conceptual understandings of “deservingness” and “difference” as portrayed through negative media and political discourse representations of European refugees [32]. These negative representations of refugees “shift[s] blame from historical, political-economic structures to the displaced people themselves […] demarcate the “deserving” refugee from the “undeserving” asylum-seeker and play into fear of cultural, religious, and ethnic difference in the midst of increasing anxiety and precarity for many in Europe” [32].

Moreover, study findings demonstrated that current migration-related policies contributed to asylum-seeker experiences of marginalization and discrimination. For instance, our study revealed that the lack of access to work translated into less social opportunities to interact with French individuals and reduced the capacity for building social cohesion. A previous study with French asylum-seekers demonstrated how if asylum-seekers were granted access to work after 1 month or less, negative attitudes between asylum-seekers and French nationals would be drastically reduced, therefore, positively impacting social cohesion [13]. These limitations imposed by macro-level legal barriers ultimately harmed asylum-seekers' capacity to flourish within their host society [73].

Asylum-seeker-related policies not only hindered asylum-seekers from flourishing in the short-term through exclusion from employment and stricter access to healthcare, but these policies also affect asylum-seekers in the long-term by creating prolonged periods of uncertainty as asylum-seekers wait in limbo for the outcomes of their asylum applications. Many asylum-seekers within our study also spoke about the inability to connect with other asylum-seekers in the long-term because after a certain period of time, they were displaced to another asylum-seeker center in a different part of the country or had to survive on their own under precarious conditions. This phenomenon is referred to as a ‘dispersion policy’ [27]. Grace et al. [30] refer to these conditions created by current policies related to the governance of migration as the “violence of uncertainty,” which undermine asylum-seekers’ mental health and well-being by creating systematic insecurity [30].

The qualitative methodology offered insight into the personal accounts and experiences regarding mental health and well-being among asylum-seekers and refugees. Additionally, a more holistic view of mental health and well-being—beyond clinical conceptualization—is considered within this study, which acknowledges the importance of flourishing in their host society. This holistic view lends itself to a more nuanced understanding of the role each social environmental factor plays in overall asylum-seeker mental health and well-being.

Further research is needed on how social networks, social support, and social cohesion impact the physical health of asylum-seekers. We also need future studies that elucidate the role of each of these factors over a longer period and under evolving political, economic, and social dynamics in France and the European Union. In addition, it is important to understand the French sociopolitical context related to asylum-seekers to interpret the study findings and policy implications. Namely, the French far right has expressed anti-asylum-seeker sentiments over the last decade following the Arab Spring Uprisings. For example, Marine Le Pen (who ran for the French presidency in 2012, 2017, and 2022) and is a member of the National Assembly of France and the National Rally, a nationalist, right-wing populist, and far-right political party in France, opposes asylum-seeker integration in order to “preserve ‘French identity’” [60]. However, even those on the left have demonstrated anti-asylum-seeker practices. In 2018, the United Nations [68] criticized France and the Macron government for “increasingly regressive migration policies and the inhumane and substandard conditions suffered by asylum-seekers” [68]. Macron’s administration was also responsible for the systematic destruction of asylum-seeker settlements by authorities in Paris and the city of Calais whose proximity to the English Channel makes it attractive for asylum-seekers trying to reach the United Kingdom.

Introducing more inclusive policies related to the governance of migration that understand asylum-seekers as “French in waiting” to borrow from Motomura’s [44] conceptualization in the United States context, is key to promoting social cohesion in France. France must embrace its state responsibility of facilitating social integration through inclusive policies at the local and federal levels. Ultimately, policies of inclusion establish a foundation for social cohesion, integration, mutual understanding, learning, and trust in a host society [50]. Additionally, from a social determinants of health perspective [64, 75]), policymakers have yet to adopt an approach that views all public policies (including im/migration policy) as health policies [16]. Civil society organizations and other non-governmental actors have played a vital role in providing different types of support to asylum-seekers. Their efforts highlight how the French government needs to adopt an intersectoral approach to the governance of migration. This perspective would contribute to the social inclusion of asylum-seekers and ultimately, increase social cohesion among all members of society.

Social inclusion has been shown to affect asylum-seeker health through participation in economic, social, and political activities [12, 15]. Overall, it is vital to recognize how more inclusive French public policies can improve the social environment and, in turn, positively affect the mental health, well-being, and the capacity of asylum-seekers to flourish in France.

## Data Availability

Data can be made available upon reasonable request.
